# Influence of metabolic syndrome on prognosis of patients with surgically treated esophageal cancer: a meta-analysis

**DOI:** 10.1186/s13098-024-01335-7

**Published:** 2024-05-23

**Authors:** Zhao Zhang, Congcong Huang, Mengshan Xu

**Affiliations:** 1 Department of Breast Oncology, Hainan Cancer Hospital, The Affiliated Cancer Hospital of Hainan Medical University, 570311 Haikou City, Hainan Province China; 2 Department of Thoracic Surgery, Hainan Cancer Hospital, The Affiliated Cancer Hospital of Hainan Medical University, No. 9, Changbin West Fourth Street, Xiuying District, 570311 Haikou City, Hainan Province China

**Keywords:** Esophageal cancer, Metabolic syndrome, Surgical resection, Survival, Meta-analysis

## Abstract

**Background:**

Metabolic syndrome (MetS) has been related to the increased incidence of esophageal cancer (EC). The aim of the study was to evaluate the influence of MetS on prognosis of patients with surgically treated EC in a systematic review and meta-analysis.

**Methods:**

An extensive search was conducted on PubMed, Embase, Web of Science, Wanfang, and CNKI to identify relevant cohort studies. Random-effects models were employed to combine the findings, taking into account the potential influence of heterogeneity.

**Results:**

Seven cohort studies involving 4332 patients with stage I-III EC who received surgical resection were included. At baseline, 608 (14.0%) patients had MetS. Pooled results suggested that MetS were associated with a higher risk of postoperative complications (risk ratio [RR]: 1.30, 95% confidence interval [CI]: 1.03 to 1.64, *p* = 0.03; I^2^ = 0%). However, the overall survival (RR: 1.07, 95% CI: 0.75 to 1.52, *p* = 0.71; I^2^ = 80%) and progression-free survival (RR: 1.27, 95% CI: 0.53 to 3.00, *p* = 0.59; I^2^ = 80%) were not significantly different between patients with and without MetS. Subgroup analyses suggested that the results were not significantly modified by study design (prospective or retrospective), histological type of EC (squamous cell carcinoma or adenocarcinoma), or diagnostic criteria for MetS (*p* values indicating subgroup difference all > 0.05).

**Conclusion:**

Although MetS may be associated with a moderately increased risk of postoperative complications in patients with EC under surgical resection, the long-term survival may not be different between patients with and without MetS.

**Supplementary Information:**

The online version contains supplementary material available at 10.1186/s13098-024-01335-7.

## Introduction

Esophageal cancer (EC) is the seventh most common cancer and the sixth leading cause of cancer-related death worldwide [1, 2]. Histologically, esophageal squamous cell carcinoma (ESCC) is the predominant type of EC in Asian patients, while esophageal adenocarcinoma (EAC) is primary type in patients from the western countries [3]. For most patients with EC, surgical resection remains the principle treatment, with the adjuvant chemo-, radio-, and immunotherapy [4, 5]. However, the prognosis of patients with EC remains poor, with current overall 5-year survival rate is approximately 20% [6]. Therefore, it is important to determine the associated clinical factors which may modify the prognosis of patients with EC.

Metabolic disorders are common in patients with various cancers. Metabolic syndrome (MetS), defined as a cluster of metabolic disorders including central obesity, insulin resistance, hypertension, and dyslipidemia, has been related to multiple chronic diseases, including cancer [7, 8]. Pathophysiologically, MetS is characterized by low-grade chronic inflammation [9], which has been also recognized as a key mechanism in carcinogenesis [10]. Accumulating evidence suggests that people with MetS may have a higher risk of EC [11]. However, most studies have been conducted in western countries, focusing on EAC [12]. Accordingly, since MetS is reversible, lifestyle changes or medical interventions targeting MetS patients might be potential prevention strategies for gastrointestinal cancers [13]. On the other hand, previous studies evaluating the influence of MetS on clinical prognosis of patients with EC showed inconsistent results [14–20]. For example, an early study including 596 patients with ESCC showed that baseline comorbidity of MetS is associated with better overall survival (OS) as compared to patients without MetS [14]. However, a subsequent study of 179 patients with ESCC suggested that MetS may be a predictor of poor OS in these patients, even after adjusting of potential confounding factors [19]. In view of this uncertainty, we performed a meta-analysis to evaluate the influence of MetS on the incidence of postoperative complications and long-term survival of patients with surgically treated EC.

## Materials and methods

The research followed the Meta-analyses Of Observational Studies in Epidemiology (MOOSE) guideline [21] and the Cochrane Handbook [22] consistently during the phase of planning, execution, and documentation.

### Inclusion and exclusion criteria of studies

The development of inclusion criteria adhered to the PICOS recommendations and aligned with the objective of the meta-analysis.

P (patients): Patients with confirmed diagnosis of EC who were treated with surgical resection.

I (exposure): Patients with MetS at baseline. The definition of MetS was in accordance with the criteria used among the included studies.

C (control): Patients without MetS at baseline.

O (outcomes): Reported at least one of the following outcomes between EC patients with and without MetS, including postoperative complications, OS, and progression-free survival (PFS). Postoperative complications were defined as adverse postoperative events of grade II or worse according to the Clavien-Dindo classification. Specifically, these complications refer to deviation from the normal postoperative course that need for pharmacological treatment (grade II), surgical, endoscopic and radiological interventions (grade III), life-threatening complications requiring intermediate care or intensive care unit-management (grade IV), and death of a patient (grade V) [23]. In addition, OS was defined as time from diagnosis to death from any cause, and PFS was defined as time from diagnosis to disease progression or relapse, unplanned re-treatment after initial management.

S (study design): Cohort studies, including prospective and retrospective cohorts.

Excluded from the meta-analysis were literature reviews, editorials, meta-analyses, and studies that including patients with other cancers rather than EC, did not assess MetS as an exposure variable, or did not report the outcomes of interest during follow-up. In instances where there was a duplication of patient populations, the study with the most extensive sample size was incorporated into the meta-analysis.

### Search of databases

Studies relevant to the objective of the meta-analysis was identified by search of electronic databases, namely PubMed, Embase, Web of Science, Wanfang, and China National Knowledge Infrastructure (CNKI) encompassing the period from inception to October 1, 2023. The search strategy employed relevant terms pertaining to the subject matter of our investigation, aiming to identify studies published within this timeframe, which included: (1) “metabolic syndrome” OR “insulin resistance syndrome” OR “syndrome X”; (2) “esophageal” OR “esophagus” OR “oesophageal” OR “oesophagus”; and (3) “carcinoma” OR “adenocarcinoma” OR “cancer” OR “tumor” OR “malignancy” OR “malignant” OR “neoplasm”. The full search strategies for each database were summarized in **Supplemental Material 1**. Only studies that met the criteria of being published as full-length articles in English or Chinese and appearing in peer-reviewed journals were included in our analysis. Additionally, during our manual screening process, we thoroughly examined the references cited in relevant original and review articles to identify any potentially relevant studies.

### Data extraction and quality evaluation

Two authors conducted literature searches, collected data, and assessed the quality of the studies separately. In instances where inconsistencies arose, the authors engaged in discussions to reach a consensus. The analysis of the studies involved gathering data pertaining to study details, design attributes, diagnosis of the patients, sample size, patient demographics, diagnostic criteria for MetS, number of patients with MetS, follow-up durations, outcomes reported, and potential confounding factors adjusted when the association between MetS and the prognosis of patients with EC was analyzed. The quality of the study was evaluated using the Newcastle-Ottawa Scale (NOS) [24]. This scale assesses the quality of cohort studies based on three dimensions: the selection of study groups, the comparability of these groups, and the ascertainment of the outcome of interest. The NOS varied between one to nine stars, with a higher star indicating a better study quality.

### Statistics

Risk ratios (RRs) and their corresponding 95% confidence intervals (CIs) were utilized as the variables to assess the relationship between MetS and prognosis outcomes in surgically treated patients with EC. In order to stabilize and standardize the variance, a logarithmic transformation was implemented on the RR and its corresponding standard error in each study [25]. The Cochrane Q test and the I^2^ statistic [26] were utilized to assess between-study heterogeneity. A value of I^2^ exceeding 50% signifies the existence of substantial heterogeneity among the studies. The random-effects model was employed for synthesizing the results, as it is acknowledged for its ability to accommodate potential heterogeneity [22]. Predefined subgroup analysis was conducted to explore whether the results were significantly modified by characteristics such as study design (prospective or retrospective), histological type of EC (squamous cell carcinoma or adenocarcinoma), and diagnostic criteria for MetS. Publication bias was estimated using a funnel plot, which involved visual assessments of symmetry, as well as Egger’s regression asymmetry test [27]. The statistical analyses were conducted using RevMan (Version 5.1; Cochrane Collaboration, Oxford, UK) and Stata software (version 12.0; Stata Corporation, College Station, TX).

## Results

### Database search and study retrieval

**Fig. 1:**
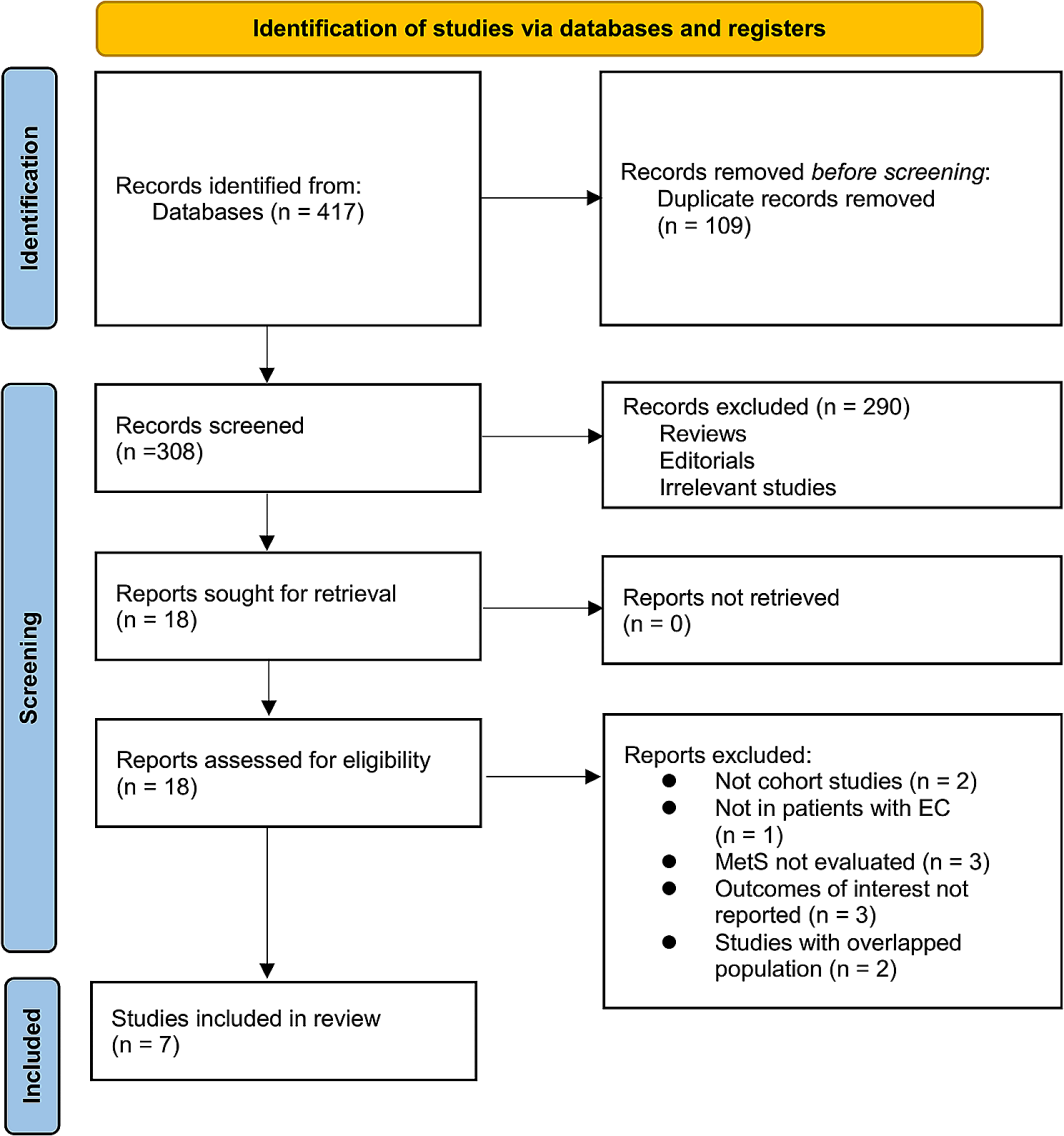
Flowchart of database search and study inclusion

Figure 1 illustrates the procedure employed for conducting the literature search and study retrieval. Initially, a total of 417 records were acquired from the designated database, and subsequently, 109 duplicate entries were eliminated. Upon scrutinizing the titles and abstracts, an additional 290 studies were excluded due to their incompatibility with the objectives of the meta-analysis. Following comprehensive evaluations of the full texts of 18 studies, 11 were excluded based on the rationales outlined in Fig. 1. Consequently, seven studies [14–20] were deemed suitable for the subsequent meta-analysis.

### Study characteristics

Overall, seven cohort studies, including three prospective cohort studies [15, 16, 20] and four retrospective cohort studies [14, 17–19] were included in the meta-analysis. The characteristic of the studies are summarized in Table 1. These studies were conducted in China and Ireland, and were published within the timeframe of 2016 to 2022. All of the included patients with stage I-III EC for surgical resection. Two studies included patients with esophageal adenocarcinoma (EAC) [15, 20] and five studies included patients with esophageal squamous cell carcinoma (ESCC) [14, 16–19]. Overall, 4332 patients with EC were included. The mean ages of the patients were 53 to 63.4 years. The diagnosis of MetS was in accordance with the National Cholesterol Education Program Adult Treatment Panel III (NCEP-ATP III) criteria in two studies [14, 20], with the International Diabetes Federation (IDF) criteria in one study [15], and with Chinese Diabetes Society (CDS) criteria in the other four studies [16–19]. At baseline, 608 (14.0%) patients had MetS accordingly. Outcomes of postoperative complications were reported in four studies [14, 15, 19, 20], OS in six studies [14, 16–20], and PFS in three studies [17–19]. Potential confounding factors such as age, sex, tumor stage, and treatments were adjusted in the multivariate analyses to a varying degree among the included studies. The NOS of these studies ranged from eight to nine, indicating their high quality (Table 2).

**Table 1:** Characteristics of the included studies

Study	Study location	Design	Diagnosis	Patient number	Mean age (years)	Male (%)	MetS diagnosis	Number of patients with MetS at baseline	Median follow-up durations (months)	Outcomes reported	Variables adjusted/matched
Wen 2016	China	RC	ESCC patients for surgical resection	596	58	73.8	NCEP-ATP III	66	42.9	POC, OS	Age, sex, smoking, alcohol consumption, tumor stage, and treatments
Doyle 2017	Ireland	PC	EAC patients for surgical resection	113	63.4	77.6	IDF	46	During hospitalization	POC	Age, sex, smoking status, ECOG and ASA grade, presence of comorbidities, and operation type
Peng 2017	China	PC	ESCC patients for surgical resection	2396	56.5	76	CDS	261	38.2	OS	Age, sex, smoking, alcohol drinking, esophagus location, histological differentiation, tumor embolus, tumor stage, tumor size
Liu 2018	China	RC	ESCC patients for surgical resection	519	62.1	81.9	CDS	53	39.6	OS, PFS	Age, sex, tumor size, tumor stage, histological differentiation, and treatments
Liu 2020	China	RC	ESCC patients for surgical resection	179	60.2	81.6	CDS	26	37	OS, PFS	Age, sex, smoking, alcohol drinking, comorbidities, tumor stage, and location
Chen 2021	China	RC	ESCC patients for surgical resection	75	53	68	CDS	22	32	OS, PFS	Age, sex, and tumor stage
Elliott 2022	Ireland	PC	EAC patients for surgical resection	454	63	87	NCEP-ATP III	134	36	POC, OS	Age, sex, ASA grade, comorbidities, and tumor stage

MetS, metabolic syndrome; RC, retrospective cohort; PC, prospective cohort; ESCC, esophageal squamous cell carcinoma; EAC, esophageal adenocarcinoma; NCEP-ATP III, National Cholesterol Education Program Adult Treatment Panel III; IDF, International Diabetes Federation; CDS, Chinese Diabetes Society; POC, postoperative complications; OS, overall survival; PFS, progression-free survival; ECOG, Eastern Cooperative Oncology Group; ASA, American Society of Anesthesiology

**Table 2:** Study quality evaluation via the Newcastle-Ottawa Scale

Study	Representativeness of the exposed cohort	Selection of the non-exposed cohort	Ascertainment of exposure	Outcome not present at baseline	Control for age and sex	Control for other confounding factors	Assessment of outcome	Enough long follow-up duration	Adequacy of follow-up of cohorts	Total
Wen 2016	1	1	1	1	1	1	1	1	1	9
Doyle 2017	1	1	1	1	1	1	1	0	1	8
Peng 2017	1	1	1	1	1	1	1	1	1	9
Liu 2018	0	1	1	1	1	1	1	1	1	8
Liu 2020	0	1	1	1	1	1	1	1	1	8
Chen 2021	0	1	1	1	1	1	1	1	1	8
Elliott 2022	1	1	1	1	1	1	1	1	1	9

### Influence of MetS on postoperative complications in patients with EC

**Fig. 2:**
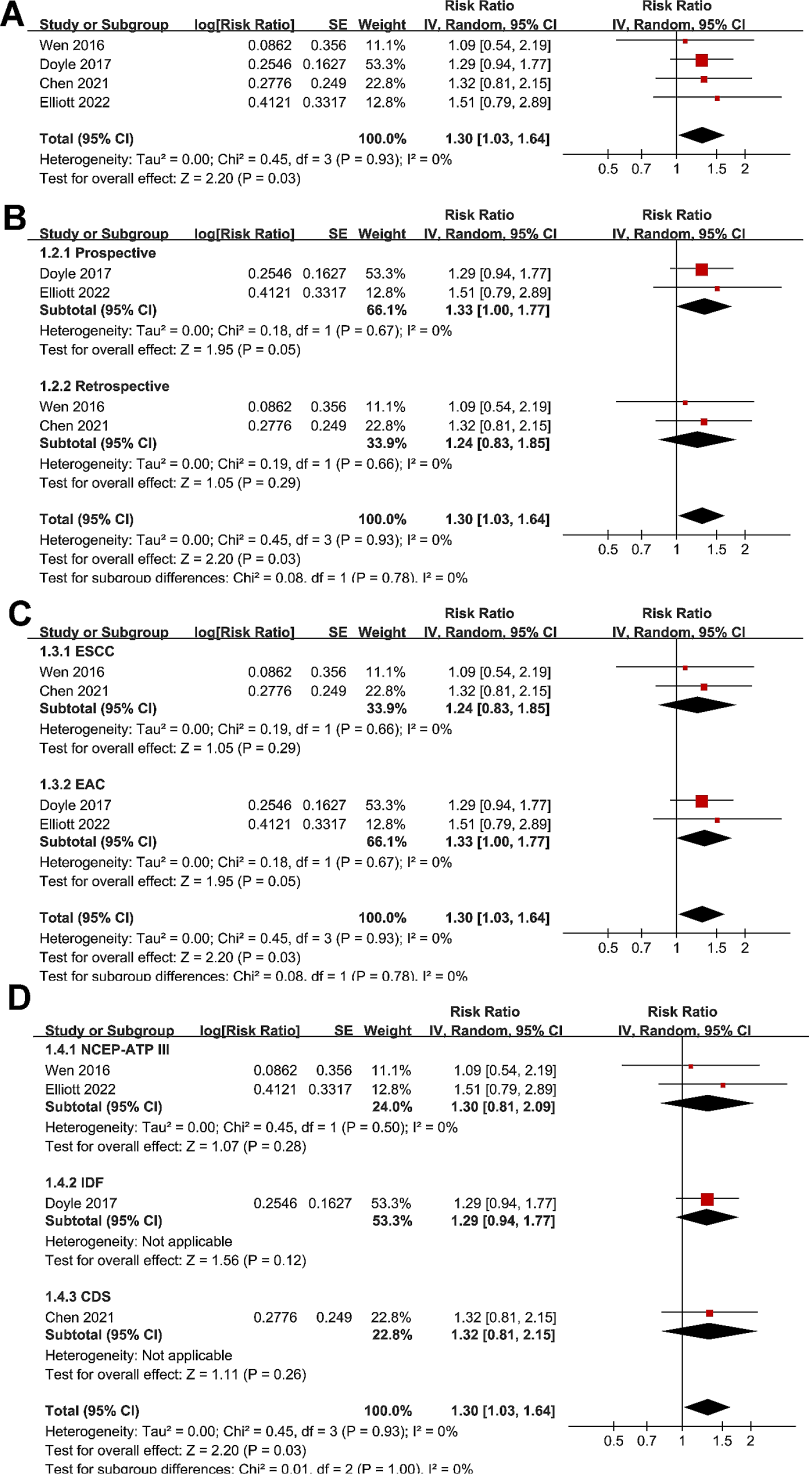
Forest plots for the meta-analysis regarding the association between MetS and the incidence of postoperative complications in EC patients after surgical resection; **A**, overall meta-analysis; **B**, subgroup analysis according to study design; **C**, subgroup analysis according to histological type; and **D**, subgroup analysis according to diagnostic criteria for MetS

Meta-analysis with four studies [14, 15, 19, 20] showed that MetS were associated with a higher risk of postoperative complications (RR: 1.30, 95% CI: 1.03 to 1.64, *p* = 0.03; I^2^ = 0%; Fig. 2A) in surgically treated patients with EC. Further subgroup analyses suggested that the results were not significantly modified by study design (p for subgroup difference = 0.78, Fig. 2B), histological type of EC (p for subgroup difference = 0.78, Fig. 2C), or diagnostic criteria for MetS (p for subgroup difference = 1.00, Fig. 2D).

### Influence of MetS on long-term survival of patients with EC

**Fig. 3:**
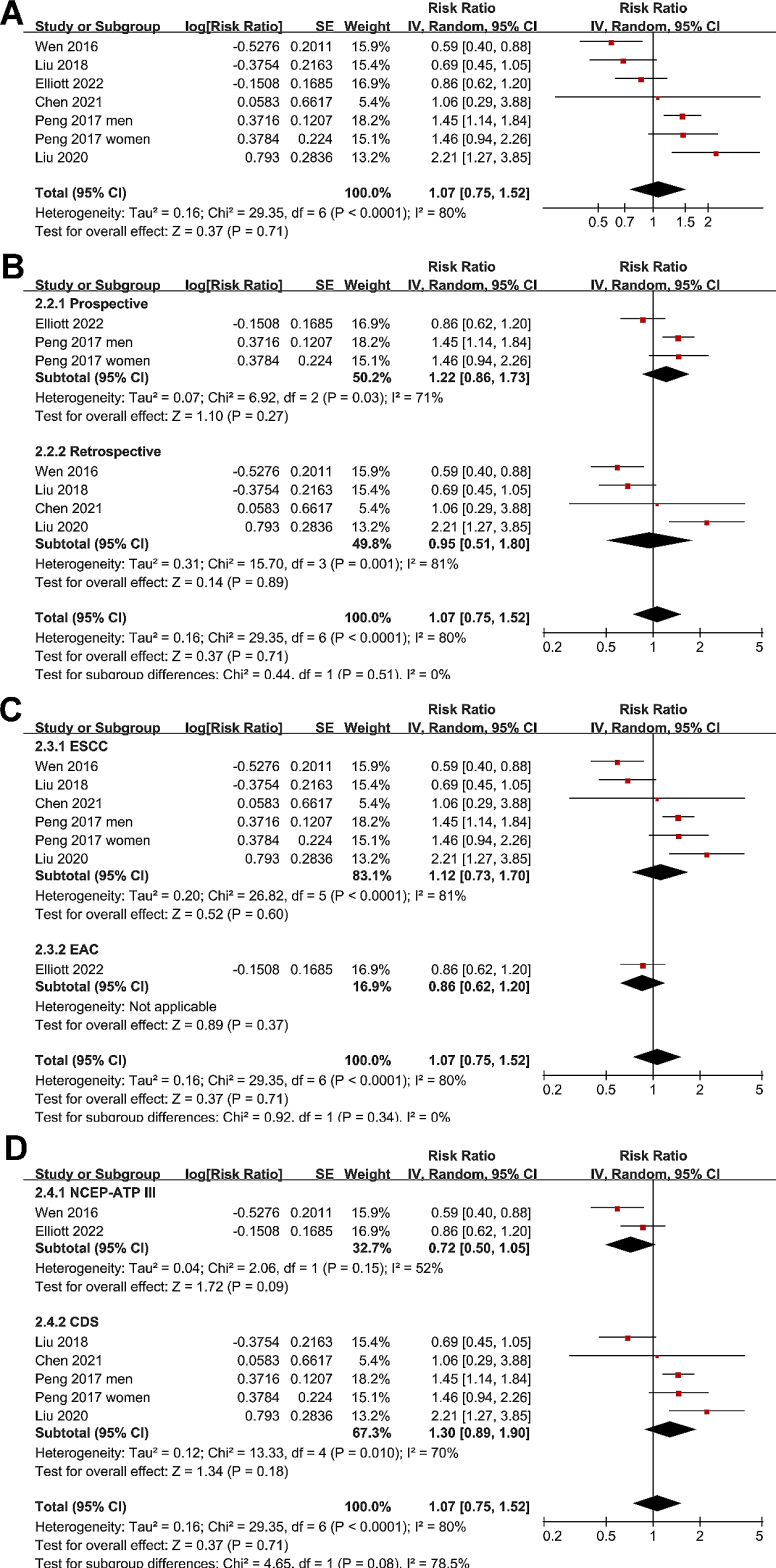
Forest plots for the meta-analysis regarding the association between MetS and OS of EC patients after surgical resection; **A**, overall meta-analysis; **B**, subgroup analysis according to study design; **C**, subgroup analysis according to histological type; and **D**, subgroup analysis according to diagnostic criteria for MetS

**Fig. 4:**
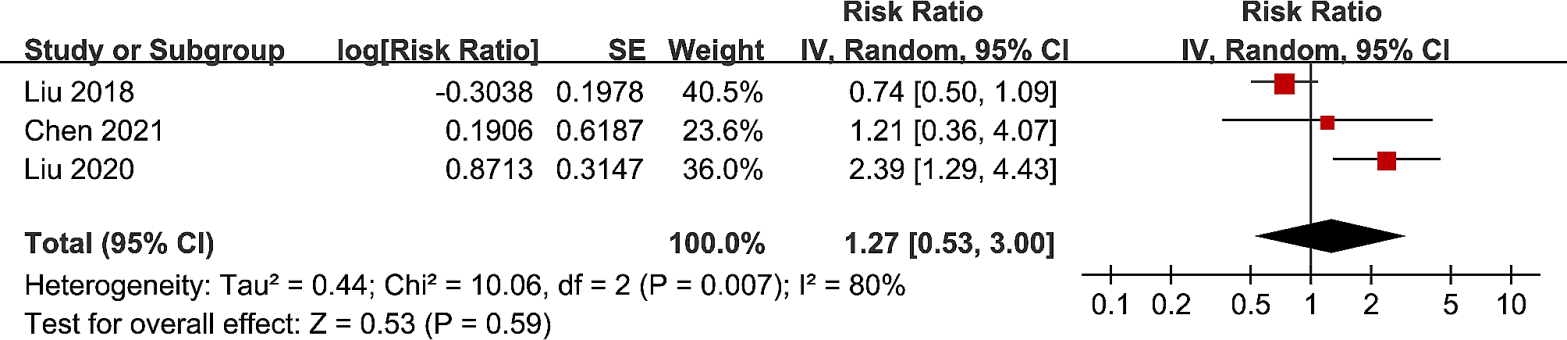
Forest plots for the meta-analysis regarding the association between MetS and PFS of EC patients after surgical resection

Since one study reported the association between MetS and OS of EC by gender, these datasets were included independently [16]. Accordingly, seven datasets from six studies [14, 16–20] reported the outcome of OS. The mean follow-up duration was 38.6 months. Results of the meta-analysis showed that the OS was not significantly different between EC patients with and without MetS (RR: 1.07, 95% CI: 0.75 to 1.52, *p* = 0.71; I^2^ = 80%; Fig. 3A). Similarly, further subgroup analyses suggested that the results were not significantly modified by study design (p for subgroup difference = 0.51, Fig. 3B), histological type of EC (p for subgroup difference = 0.34, Fig. 3C), or diagnostic criteria for MetS (p for subgroup difference = 0.08, Fig. 3D). In addition, three studies reported the outcome of PFS [17–19], with the mean follow-up duration of 38.3 months. All of the three studies were of retrospective design, included patients of ESCC, and used CDS criteria for the diagnosis of MetS. Pooled results of the three studies suggested that MetS was not significantly associated with PFS in EC patients after surgical resection (RR: 1.27, 95% CI: 0.53 to 3.00, *p* = 0.59; I^2^ = 80%; Fig. 4).

### Publication bias

**Fig. 5:**
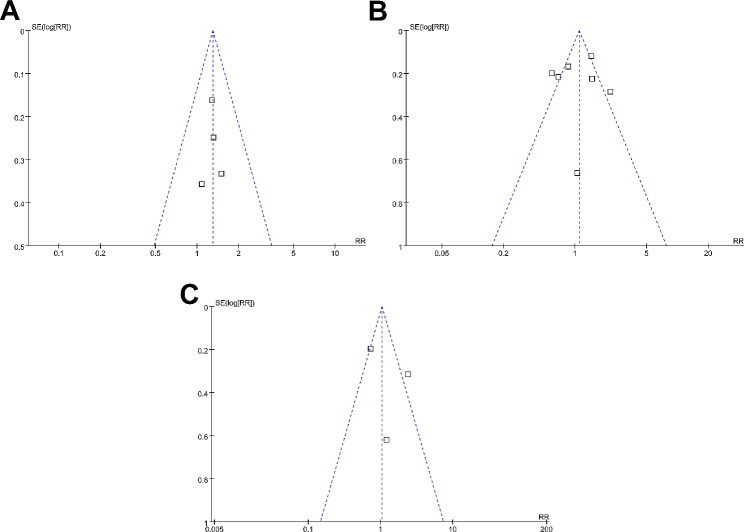
Funnel plots for the publication biases underlying the meta-analyses; **A**, funnel plots for the outcome of postoperative complications; **B**, funnel plots for the outcome of long-term OS; and **C**, funnel plots for the outcome of long-term PFS

The funnel plots depicting the meta-analyses of the associations between MetS with postoperative complications, OS, and PFS of surgically treated patients with EC are presented in Fig. 5A and C. Upon visual inspection, the plots exhibit symmetrical patterns, indicating a minimal presence of publication bias. The Egger’s regression tests were not performed because limited datasets were incorporated for each outcome.

## Discussion

In this study, we pooled the results of seven eligible cohort studies, and the results showed that MetS was associated with a higher incidence of postoperative complications in patients with surgically treated EC. However, subsequent meta-analyses did not show a significant difference of OS and PFS between EC patients with and without MetS at baseline. Further subgroup analysis suggested that the results may not be significantly affected by study design, histological type of EC, and diagnostic criteria for MetS. Taken together, results of the meta-analysis suggested that although MetS may be associated with a moderately increased risk of postoperative complications in patients with EC under surgical resection, the long-term survival may not be different between patients with and without MetS.

To the best of our knowledge, no previous meta-analysis has investigated the potential influence of MetS on prognosis of patients with EC. In this meta-analysis, we focused on EC patients with surgical treatment, and an extensive literature search was performed in five commonly used electronic databases, which retrieved seven up-to-date cohort studies. Only cohort studies were considered in this meta-analysis, aiming to evaluate the longitudinal relationship between MetS and prognosis of patients with EC. Multivariate analyses were used in all of the included studies when the association between MetS and prognostic outcomes of EC were analyzed, which therefore could minimize the influence of potential confounding factors. Finally, a series of predefined subgroup analyses were performed to evaluate the robustness of the findings, which suggested that the results were not significantly modified by study design, histological type of EC, or definition of MetS.

Overall, we found that a short-term influence of MetS on the risk of postoperative complications in patients with surgically treated EC. These findings are similar to findings of previous studies in patients with digestive system malignancies of other sites. An early meta-analysis included six studies and showed that MetS may have a negative impact on adverse outcome after colorectal surgery, such as the increased risk of anastomotic leakage [28]. A more recent meta-analysis in patients with gastric cancer also showed that MetS was associated with higher risks of postoperative complications [29]. Similar results were also observed in a recent large-scale cohort study, which showed that MetS was associated with a higher risk of postoperative complications in patients with hepatocellular carcinoma after hepatectomy [30]. These findings suggest that in short-term, MetS may be a risk factor for the postoperative complications in patients receiving surgical treatment for EC. The mechanisms may be related to the chronic systematic inflammation in patients with MetS. For example, compared to patients without MetS, patients with EC and MetS were shown to have higher perioperative circulating C-reactive protein (CRP), suggesting an activated inflammatory response [15]. Previous studies have confirmed that a higher level of CRP in patients receiving esophagectomy for EC was related to a higher risk of postoperative complications [31], such as anastomotic leakage [32] and postoperative pneumonia [33], which may be an explanation between MetS and increased risk of postoperative complications in these patients.

On the other hand, results of the meta-analysis failed to show that MetS may adversely affect the long-term survival of patients with EC. These findings may be explained by the potential different influences of the components of MetS on the prognosis of patients with EC. For example, obese and overweight patients with EC were shown to have a more favorable long-term survival than patients with normal weight [34]. Similarly, a higher post-treatment serum triglyceride has also been suggested as a predictor of favorable overall survival [35]. Although postoperative hyperglycemia was shown to adversely affect the survival of non-diabetic patients with EC after surgery [36], a previous meta-analysis suggested that diabetes may have no significant impact on long-term survival of EC patients who undergo esophagectomy [37]. As for hypertension, an early cohort study suggested that hypertension may be a predictor of poor survival in patients with ESCC after esophagectomy [38], while subsequent studies showed that the results may be different according to antihypertensive drugs used [39, 40]. Collectively, current evidence did not support that MetS may significantly affect the long-term survival of patients with EC after esophagectomy. The interactions between MetS and long-term survival of EC are complicated, which are depending on the influences of the components of MetS and related treatments for these metabolic disorders.

This study has limitations. First, the protocol of the meta-analysis was not registered prospectively on any online registration website. Second, the number of available datasets for the meta-analysis is limited, and the results should be better validated in large-scale prospective studies. Third, although multivariate analyses were used among the original studies to estimate the relationship between MetS and prognostic outcomes of patients with EC, there may be residual unadjusted factors which may modify the results. For example, we could not determine the concomitant adjuvant anticancer treatment on the results of the meta-analysis because these variables were largely not reported among the included studies. Moreover, although we found that EC patients with MetS may be associated with an increased risk of overall postoperative complications, the influence of MetS on individual postoperative adverse events is still not known. In addition, we included studies of different diagnostic criteria for MetS, which may influence the results of the meta-analysis and cause between-study heterogeneity. Large-scale prospective studies are encouraged to investigate the different diagnostic criteria for MetS could significantly affect the association between MetS and prognosis of patients with EC. Finally, it may be more clinically relevant to determine the influences of interventions for each component of MetS on the postoperative complications and survival of patients with EC. Further studies are warranted in the future.

## Conclusions

In conclusion, results of the meta-analysis indicate that although MetS may be associated with a moderately increased risk of postoperative complications in patients with EC under surgical resection, the long-term survival may not be different between patients with and without MetS. Although these findings should be validated in large-scale prospective studies, the results suggest that the influences of MetS on long-term survival of patients with EC may be complicated, depending on the individual components of MetS and associated interventions for these metabolic disorders.

### Electronic supplementary material

Below is the link to the electronic supplementary material.

Supplementary Material 1

## Data Availability

All data generated or analyzed during this study are included in this published article.
