# Supplementary material for: Influence of metabolic syndrome on prognosis of patients with surgically treated esophageal cancer: a meta-analysis
Source: Diabetol Metab Syndr. 2024 May 23;16:111. doi: 10.1186/s13098-024-01335-7 (PMC11112923; doi:10.1186/s13098-024-01335-7)
Supplement: Supplementary file 1 — Supplementary Material 1 [file 13098_2024_1335_MOESM1_ESM.docx]

**Keywords based search strategy for PubMed, Embase, and Web of Science**

("metabolic syndrome" OR "insulin resistance syndrome" OR "syndrome X") AND ("esophageal" OR "esophagus" OR "oesophageal" OR "oesophagus") AND ("carcinoma" OR "adenocarcinoma" OR "cancer" OR "tumor" OR "malignancy" OR "malignant" OR "neoplasm")

**Keywords based search strategy for Wanfang and CNKI**

“代谢综合征” AND “食管癌”
